# Pangenome Data Analysis Reveals Characteristics of Resistance Gene Analogs Associated with *Sclerotinia sclerotiorum* Resistance in Sunflower

**DOI:** 10.3390/life14101322

**Published:** 2024-10-17

**Authors:** Yan Lu, Jiaying Huang, Dongqi Liu, Xiangjiu Kong, Yang Song, Lan Jing

**Affiliations:** College of Horticulture and Plant Protection, Inner Mongolia Agricultural University, Huhhot 010011, China; luyan820918@126.com (Y.L.); jyhuang666@126.com (J.H.); liudongqi12306@126.com (D.L.); xjkong0528@126.com (X.K.); dbmsy@sina.cn (Y.S.)

**Keywords:** sunflower, pangenome, RGAugury, resistance gene, *Sclerotinia sclerotiorum*

## Abstract

The sunflower, an important oilseed crop and food source across the world, is susceptible to several pathogens, which cause severe losses in sunflower production. The utilization of genetic resistance is the most economical, effective measure to prevent infectious diseases. Based on the sunflower pangenome, in this study, we explored the variability of resistance gene analogs (RGAs) within the species. According to a comparative analysis of RGA candidates in the sunflower pangenome using the RGAugury pipeline, a total of 1344 RGAs were identified, comprising 1107 conserved, 199 varied, and 38 rare RGAs. We also identified RGAs associated with resistance against *Sclerotinia sclerotiorum* (*S. sclerotiorum*) in sunflower at the quantitative trait locus (QTL). A total of 61 RGAs were found to be located at four quantitative trait loci (QTLs). Through a detailed expression analysis of RGAs in one susceptible and two tolerant sunflower inbred lines (ILs) across various time points post inoculation, we discovered that 348 RGAs exhibited differential expression in response to Sclerotinia head rot (SHR), with 17 of these differentially expressed RGAs being situated within the QTL regions. In addition, 15 RGA candidates had gene introgression. Our data provide a better understanding of RGAs, which facilitate genomics-based improvements in disease resistance in sunflower.

## 1. Introduction

The sunflower (*Helianthus annuus* L.) is one of the four most important oilseed crops in the world [1]. However, sunflower production is compromised by infection from several fungal diseases, including rust, Sclerotinia head rot (SHR), Sclerotinia stalk rot (SSR), and wilt. These diseases pose a significant threat to both yield and quality across all major sunflower growing regions [2,3,4]. Normally, the application of chemical fungicides can obviously inhibit the diseases but poses hazardous risks to human health and the environment [5]. Unlike other pathogens, *S. sclerotiorum* is necrotrophic and infects sunflowers through wounds [6]. Sclerotinia head rot is directly linked to physical injury of heads, and, as a consequence, any attempts to minimize head damage are important. However, controlling hail, birds, and insects is almost impossible. Thus, the development of genetically resistant sunflower varieties might be the most viable and sustainable strategy for disease control [7,8].

In their evolutionary arms race with pathogens, plants have acquired complicated defense mechanisms to recognize different stresses, which initiate appropriate signal transduction pathways [9,10,11]. Plants mainly protect themselves from pathogens through two layers of the biochemical immune system [12]. The first layer is implemented by the cell surface pattern recognition receptor (PRR), which detects the general inducer pathogen/microbe-associated molecular pattern (PAMP/MAMP), initiating PAMP-triggered immunity (PTI) [13]. Additionally, pathogen effectors that bind the nucleotide-binding site leucine-rich repeat (NBS-LRR) receptors activate a robust counter-defense known as effector-triggered immunity (ETI) [14]. The synergistic action of PTI and ETI can enhance a plant’s immunity and has an even more potent immune effect than independent action [14,15,16,17,18,19]. Resistance gene analogues (RGAs) play a key role in plant resistance, with quantitative and qualitative characteristics [12]. Quantitative resistance genes commonly contribute to a PTI response, while qualitative resistance genes usually cause an ETI response. Genomic studies have identified RGAs that often bear the conserved domains and motifs essential for pathogen recognition and defense response activation [20]. R products have effector recognition receptors and trigger downstream signaling during plant disease resistance [15,21]. Among these, NBS-LRR proteins, which include a nucleotide-binding site (NBS) and a C-terminal leucine-rich repeat (LRR) domain, represent a major type of receptor [22]. Based on the N-terminus motif, NLRs are categorized into three subclasses, namely TIR-NBS-LRR (TNL), which N-terminal domain with Toll/Interleukin-1 receptor homology, nucleotide binding and leucine rich repeat domains, CC-NBS-LRR (CNL), contain Coiled Coil nucleotide-binding site and a C-terminal leucine-rich repeat, and RPW8-NBS-LRR (RNL), contains the RESISTANCE TO POWDERY MILDEW8 (RPW8) domain [23]. The PRR is another kind of RGA, which is the main substance of plant immune function, including receptor-like kinases (RLKs) and receptor-like proteins (RLPs) [12,24]. RLKs are the most abundant RGAs in plants, and their structure is very similar to that of RLPs. They have an extracellular domain at the beginning of the N-terminal, which is involved in the perception of microbial patterns, and a transmembrane helical domain, which anchors RLP and RLK in the plasma membrane. Unlike RLPs, RLKs possess a C-terminal cytoplasmic kinase domain, such as Ser/Thr protein kinase or Tyr kinase (STTK), which is absent in RLPs [12,25,26]. Plant PRRs are classified according to their extracellular N-terminal domains. The major PRR subclasses involved in pathogen recognition have Lys motifs or LRR domains [27]. RLPs do not have any signaling domain in their intracellular region, and their domain structure is similar to that of the extracellular domain of RLKs, suggesting that they may function with one or more RLKs [23,26]. Beyond their role in defense, RLKs and RLPs are implicated in various developmental processes, including the formation of meristem and stomatal development [12,28,29].

To date, several resistance genes and QTLs have been reported against *Pucinia helianthi* (*P. helianthi*) or *S. sclerotiorum* in sunflower [30,31,32,33,34,35,36,37,38,39,40,41,42,43,44,45,46,47,48,49]; most studies have focused on NBS analysis. However, resistance gene analogs (RGAs) in sunflower have not been reported. Traditional gene family analyses have relied on a single reference genome, which may not include the genetic diversity present within a species. This approach risks overlooking significant genes that contribute to disease resistance, as individual plants have evolved unique genetic traits in response to environmental pressures. The concept of a pangenome encompasses the entire gene repertoire of a species, including both the reference genome and the genetic variation found among different varieties. Pangenomic analysis offers a more holistic view of a species’ genetic makeup, enabling the study of immune receptors and core signaling pathways on a broader scale. Such analysis has the potential to reveal elite gene sets that enhance plant immunity against rapidly evolving pathogens. In 2019, Hübner et al. constructed a sunflower pangenome based on the reference genome of HA412-Ho.v.1.1, revealing the abundant presence/absence variant in sunflower genes, which laid a foundation for subsequent studies [50].

In this study, we employed the RGAugury pipeline to identify and characterize RGAs from the sunflower pangenome data. We detected the presence/absence variation of RGAs and analyzed its features, such as RGA numbers, distribution, variation, and physical locations in sunflower. Normally, these RGAs appeared in a clustered way and are more likely to be lost or conserved [51]. We then investigated specific RGAs in the QTLs showing resistance against *S. sclerotiorum* through analysis. In addition, we compared and analyzed the significant differential RGAs’ expression response to *S. sclerotiorum*. To understand the population structure and evolution of different sunflower cultivars, the functional haplotype diversity of RGAs was also evaluated. The RGA study of the global pangenome might be applied to understand disease resistance and facilitate the discovery of candidate R genes for sunflower breeding programs in the future.

## 2. Materials and Methods

### 2.1. Identification of RGA Genes from Sunflower Pangenome Data

The pangenomic protein sequences of sunflower were downloaded from https://sunflowergenome.org/ (accessed on 1 May 2022). The sunflower pangenome includes 290 sunflower accessions, which captures about 90% of the allelic diversity in the cultivated sunflower gene pool. Information on the initial characterization of the sunflower pangenome can be found in Hübner et al. [50]. The RGAugury pipeline (version 2017-10-21) [52] was used to identify RGAs. Three main classes of RGAs were identified and classified as RLK, RLP, and NLR genes. The RGAugury pipeline also divided the NLR gene family members into several subgroups, namely NBS, CNL, TNL, TN, CN, NL, TX, and other candidate genes. These resistance genes were classified based on the presence or absence of specific domains. Proteins carrying only an NB-ARC domain were classified as NBS; proteins carrying TIR, NB-ARC, and leucine-rich repeat domains were classified as TNLs, or TN if the leucine-rich repeat domain was missing. Proteins carrying coils, NB-ARC, and leucine-rich repeat domains were classified as CNLs, or CN if the leucine-rich repeat domain was missing, or NL if the coils domain was missing. Proteins carrying a TIR domain with additionally unknown domains were classified as TX, while proteins carrying TIR and coils but not NB-ARC domains were classified as OTHER. Proteins carrying the RPW8 domains were manually reclassified based on their original domains. Proteins carrying NBS and RPW8 domains were classified as RN. Proteins carrying NL and RPW8 domains were classified as RNL. All other combinations (CNL + RPW8, TNL + RPW8, CN + RPW8, TN + RPW8, TX + RPW8) were classified as OTHER. Among NLRs, TNL, CNL, and RNL subgroups were named as “typical” NLRs, and the other subgroups that contain partial or disordered domains were named as “atypical” NLRs. Using a Python script (Python 3.10.4, http://www.python.org), RLKs and RLPs were divided into three subclasses: PRRs containing an LRR domain (LRR-RLK/RLP), PRRs containing lysine motifs (LysM-RLK/RLP), and PRRs with any other domain (other-RLK/RLP) [53].

### 2.2. Chromosomal Location of the RGA Gene Family in Sunflower

The location of RGAs on chromosomes was plotted using the r tracklayer package, karyoploteR package, and R ColorBrewer package in Rscript (V4.0.3).

The r tracklayer package (https://rdrr.io/bioc/rtracklayer/, accessed on 1 May 2022) was used to transform the data format. The Rpackage karyotypeR v1.2.2 (https://github.com/bernatgel/karyoploteR, accessed on 1 May 2022) was used to plot gene densities. The RColorBrewer package (https://cran.r-project.org/web/packages/RColorBrewer/RColorBrewer.pdf, accessed on 1 May 2022) was used to color palettes for maps.

### 2.3. Disease Resistance-Linked QTLs Analysis

Known blackleg resistance-linked QTLs were collected from the sunflower genome database https://sunflowergenome.org/pangenome-data/HelianthusVariants.vcf.gz (accessed on 1 May 2022) and marker sequences were collected from Talukder et al. (2016) [51]. BLAST blastn (task: blastn-short, evalue: 0.05) [54] was used to assign positions for the forward and reverse primer sequences in the v1.1 H. annuus assembly. Waterfall plots were drawn using GenVisRv1.11.3 [55], vcftools v0.1.15 [56], R version 3.4.4 (R Core Team, Vienna, Austria), and Variant Effect Predictor v88.13 [57], which was used to annotate the variation of RGA.

### 2.4. CDS Haplotype Analysis of RGAs

The sunflower variant call format (VCF) files were filtered using VCFtools, and rare alleles with a minor allele frequency of <0.001 or a missing rate of >0.4 were removed.

Gatk_vcf_to_haplotype.pl (https://github.com/zhuochenbioinfo/VCF2HAP, accessed on 1 May 2022) was used for constructing the Gene-coding sequence (CDS)-haplotype (gcHap) of the full dataset of SNPs. The SNPs were previously predicted by Hübner et al. (2019) [50]. Finally, we built a custom 3KRG gcHap dataset that was used for calculating the number of gcHaps of different populations.

Shannon’s equitability (*E_H_*) was estimated using the gcHap data to make the gene diversity among different populations comparable [58]. The formula is

$$E_{H}=\frac{1}{\ln N}\;(-\sum pi\;ln\;pi).$$

where *pi* is the proportion of the ith gcHap of a gene, *N* is the population size, and ln*N* is the maximum possible diversity of a gene. *E_H_* therefore has a value between 0 and 1. For each gene, Nei’s genetic identity (*I_Nei_*) [59] between two populations was estimated with the gcHap data and used to measure the genetic differentiation among populations. It was estimated as

$$I_{\mathrm{Nei}}=\;\frac{\sum {\;X}_{i}Y_{i}}{\sqrt{\sum {\;X}_{i}^{2}{\;Y}_{i}^{2}}}$$

where *X_i_* and *Y_i_* are the frequencies of the ith gcHap of a gene in populations *X* and *Y*, respectively. Finally, we used the R (v 4.0.3) ggplot2 package and ggpubr package to show *E_H_* and *I_Nei_* with visual graphics [60].

### 2.5. RGA Analysis of Different Sunflower Cultivars from RNA-Seq Data

To investigate RGAs in different sunflower cultivars, RNA-seq data were downloaded from NCBI, with the SRA accession number SRP219154 (https://www.ncbi.nlm.nih.gov/sra/?term=SRP219154, accessed on 1 May 2022). For the original RNA-seq data, we used fastq-dump of SRA Toolkit v2.10.0 (http://www.ncbi.nlm.nih.gov/Traces/sra/sra.cgi?view=toolkit_doc&f=fastq-dump, accessed on 1 May 2022) to convert SRA files to FASTQ format data, then removed adaptors and low-quality sequences with Fastp v0.20.1.

The RNA-Seq clean data of each sample were aligned with the sunflower pangenome using HiSAT2 V2.1.0. FPKM values of genes in all samples were calculated as expression levels. DESeq2 V1.32.0 was used for differential expression analysis. Unigenes and |log2 ratio| ≥ 1 and q < 0.05 were used as the cutoff value for significant differential expression. The converted log2 (FPKM + 1) values were used to generate heat maps using R (version 4.0.3) and the ComplexHeatmap package (v.2.6.2, https://bioconductor.org/packages/release/bioc/html/ComplexHeatmap.html, accessed on 1 May 2022).

We analyzed the diferential expression of RGA genes in each inbred line (IL)-time point combination.

### 2.6. Validation of RNA-Seq Analysis by qPCR

The susceptible cultivar HA89 and disease-tolerant cultivars HA853 and HA416 were used in this study. To evaluate the levels of the RGA genes expression of sunflower under *S. sclerotiorum* infection, three genes, including *Ha16_00022752*, *Ha16_00022056*, and *Ha16_00022401*, were selected in this experiment. Sunflower plants were grown in a greenhouse controlled at 22 °C with a 16 h light/8 h dark cycle. The 4-week-old seedlings were used for subsequent assays. One strain of *S. sclerotiorum* was supplied by College of Plant Protection, Inner Mongolia Agricultural University (Huhhot, China). *S. sclerotiorum* inoculation was performed as described by Chen et al. (2022) [61]. The tissues were collected at 0 h, 4 h, and 8 h after treatments.

Total RNA was isolated using Plant total RNA Isolation Kit (Sangon, Shanghai, China) according to the manufacturer’s instructions. Quantitative real-time RT-PCR (qPCR) was performed on an ABI Prism 7000 Sequence Detection System (PE Applied Biosystems, Foster City, CA, USA) using iQ SYBR Green supermix (Bio-Rad, Billerica, MA, USA) according to the manufacturer’s protocol. Actin and tubulin were used as endogenous reference genes. The relative expression of each selected gene was determined using the “RT-PCR comparison of relative gene expressions analysis” included in the InfoStat sofware (version 2014). The gene-specific primers are listed in Table S1.

### 2.7. Gene Ontology (GO) Annotation and Enrichment Analysis

All RGA sequences were compared with sequences in the UniPROT database using BLASTP with a 1E-5 threshold. The Retrieve/ID mapping tool was used for GO enrichment analysis. All the genes in the sunflower pangenome were used as the background genome. Cytoscape (V3.8.0) was used to demonstrate the enrichment results in the signaling pathways of the biological process, cellular component, and molecular function.

## 3. Results

### 3.1. Genome-Wide Analysis of RGA Candidates from the Sunflower Pangenome Data

**Figure 1:**
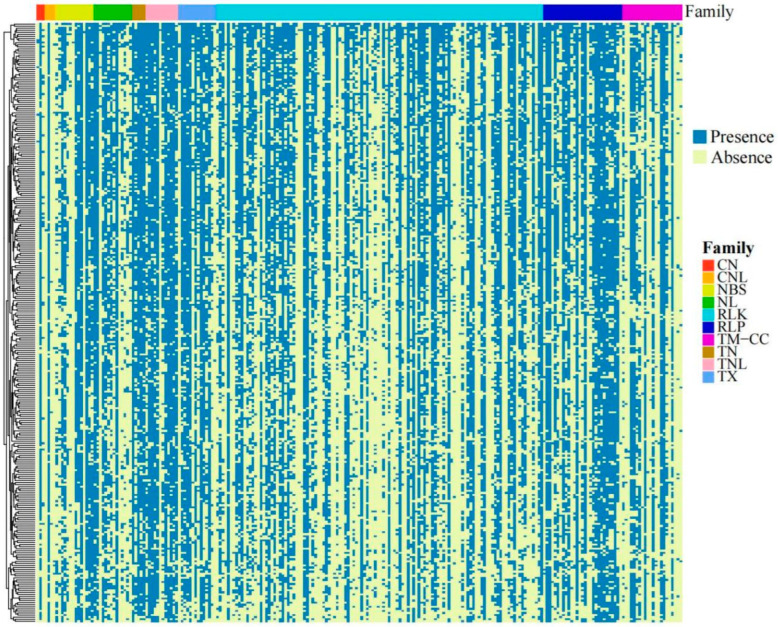
Heat maps of 10 RGA subgroups presence/absence variation (PAV) in 290 sunflower accessions. Red box indicate Coiled Coil and nucleotide-binding site (CN); orange box indicate Coiled Coil, nucleotide-binding site and a C-terminal leucine-rich repeat (CNL); yellow box indicate nucleotide-binding site (NBS); green box indicate leucine-rich repeat (NL); light blue box indicate receptor-like kinase (RLK); dark blue box indicate receptor-like proteins (RLP); purple box indicate transmembrane-coiled-coil (TM-CC); brown box indicate N-terminal domain with Toll/Interleukin-1 receptor homology and nucleotide-binding site leucine-rich repeat (TIR-NBS, TN); pink box indicate N-terminal domain with Toll/Interleukin-1 receptor homology, nucleotide binding and leucine rich repeat domains (TIR-NBS-LRR, TNL); violet box indicate TIR with unclassified domains (TX).

A total of 1344 RGAs were identified in the sunflower pangenome (Table 1 and Table S2). Of the 1344 genes, 1107 (82.4%) were core and 237 (17.6%) were variable. In total, 1331 RGAs were identified on the reference genome (230 core and 1101 variable) and 13 RGAs (7 core and 6 variable) were found in the additional contigs in the pangenome. Of the 1331 RGAs identified on the reference genome, RLK (757) was the largest class of RGA candidates, followed by TM-CC (191). Additionally, 13 RGAs, which comprise 7 core and 6 variable genes, were discovered within the additional contigs of the pangenome, not present in the reference genome assembly. These encompass 5 RLK genes, 5 TX genes, and 3 CNL genes. We compared the counts of RGA candidates within the 290 lines based on the presence/absence results (Figure 1, Table S3). A total of 32887 RGAs were identified in 290 lines, with an average of 113 RGAs per line. The range of RGAs per line varied, such as MexCult15 having the fewest at 60 RGAs and PPN226 having the most at 173 RGAs. The gene Ha14_00019475 was the most prevalent, appearing in 273 lines. The total number of different RGAs across the 290 lines on the reference genome and the pangenome additional contigs are presented in Table 2.

Within the reference genome, the 290 lines contained a total of 32,445 RGAs, with an average of 111.8 RGAs per line, 60 RGAs in MexCult15, and 171 RGAs in PPN226. In contrast, the additional pangenome contigs harbored 442 RGAs, distributed among 268 lines, with no RGAs detected in the remaining 22 lines. Notably, there were no CN, NBS, NL, TNL, TX, and RLP genes in the pangenome additional contigs.

More RLK genes (45.68%) were predicted than CNL genes (0.86%). The remaining RGAs represented 54.31%. The number of different RGA candidates and subfamilies found on the reference genome are presented in Table 2.

TM-CC showed the highest percentage of variance (88.5%), followed by RLK (84.1%), and NBS-LRR showed the lowest percentage of variable genes (75.1%). This high variability suggests that many of these genes may be pseudogenes, which the genome can tolerate losing without significant impact on resistance capabilities.

### 3.2. Distribution of RGAs on Sunflower Chromosomes

**Figure 2:**
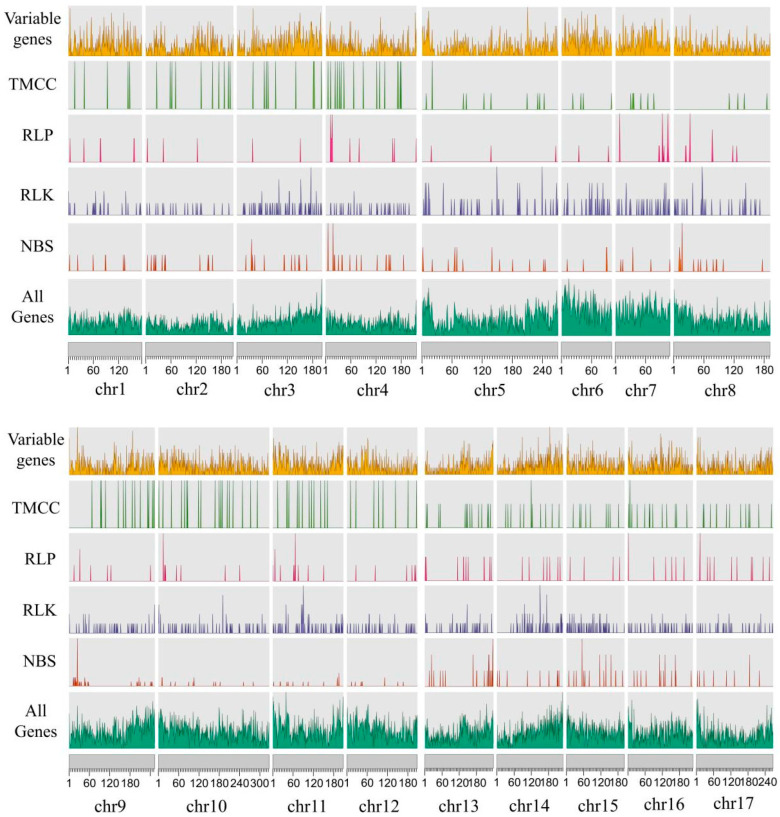
Distribution of variable genes and nucleotide-binding site (NBS), receptor-like protein (RLP), receptor-like protein kinase (RLK), and transmembrane coiled-coil domain protein (TM-CC) domains across the reference genomes.

To further explore the distribution of RGAs on chromosomes, we mapped the selected RGAs to the chromosomes of the sunflower reference genome (Figure 2). Overall, four classes of RGAs (RLK, RLP, NBS, and TM-CC) were unevenly distributed along the chromosomes. The RLKs are relatively evenly dispersed, whereas RLPs exhibit the most scattered distribution pattern among the chromosomes. Forty-four NBS-encoding genes were identified on chromosome 9 ranging from 20 MB to 30 MB. All chromosomes contained a similar number of TM-CC genes, and chromosome 10 showed the highest count (total of 19).

### 3.3. Analysis of Known Disease Resistance-Linked QTLs

It has been reported that RGA plays an important role in disease defense. We compared the RGA candidate positions with known quantitative trait loci (QTLs) for Sclerotinia stalk rot (SSR) resistance to assess possible biological functions. We located 61 QTL markers through genetic mapping across 4 loci on 4 pseudomolecules (Table 3). A total of 61 RGAs were identified within these QTLs. *Qbsr-4.1* was localized within an interval of 0.17 Mbp containing five RGAs. *Qbsr-9.1* was localized within an interval of 0.18 Mbp containing five RGAs. *Qbsr-11.1* was placed within an interval of 0.15 Mbp containing one RGA. *Qbsr-16.1* was localized within an interval of 1.54 Mbp containing 50 RGAs. The QTL names and references are shown in Table 3.

**Figure 3:**
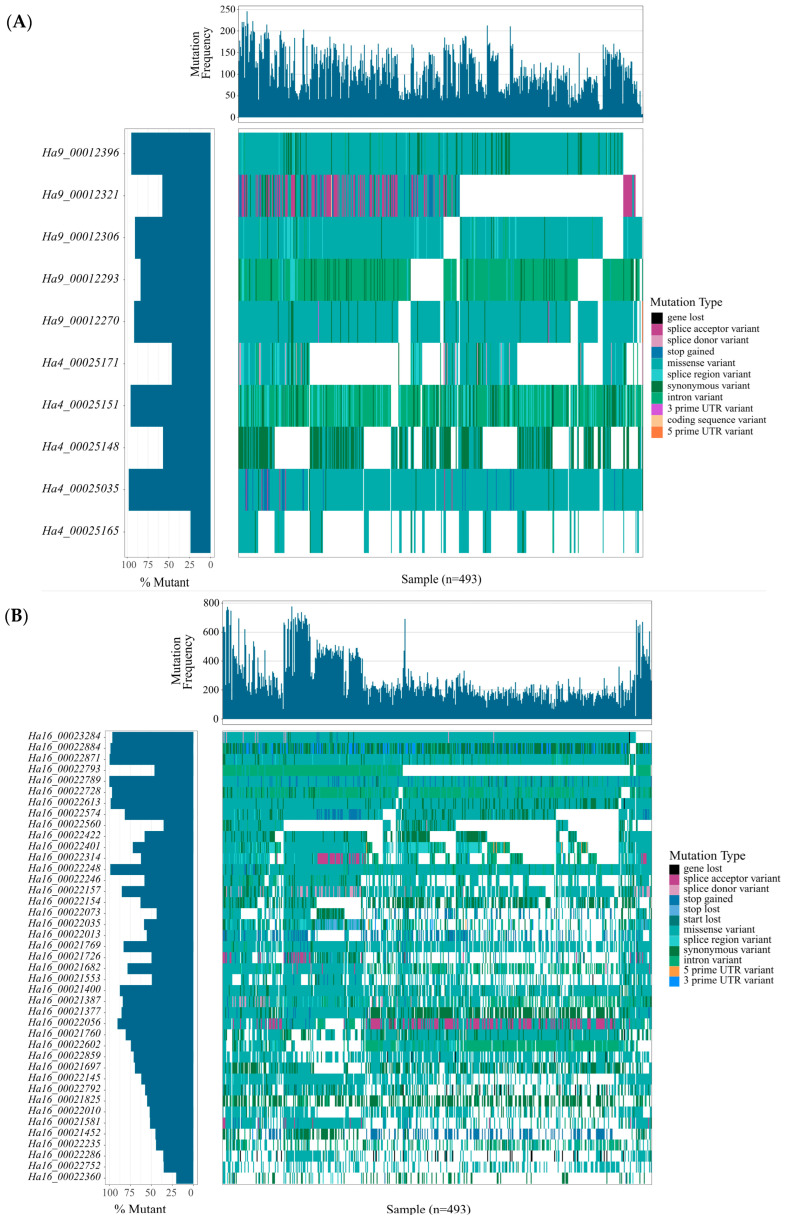
Plot of Sclerotinia resistance-linked QTL in 493 sunflower accessions: (**A**) plot of Sclerotinia resistance-linked QTLs of *Qbsr-4.1*, *Qbsr-9.1*, and *Qbsr-11.1*; (**B**) plot of Sclerotinia resistance-linked QTL of *Qbsr-16.1*. Gene order is determined by the position in the reference assembly.

To examine the variation of 61 RGAs in 493 individuals, we assessed the variation generated by SNPs and PAVs in the sunflower pangenome data (Figure 3A,B). In total, 9 of the 61 RGAs contained no SNPs, indicating that these resistance genes show stable inheritance. Thus, these nine genes are not shown in the waterfall plot. A waterfall plot of the Sclerotinia stalk rot resistance-linked QTL *QBSR-16.1* was produced to show the mutational load of RGA candidates located within the QTL candidate region in all 493 individuals (Figure 3B). *Ha4_00025035* showed the maximum mutation percentages in the Qbsr-4.1, Qbsr-9.1, and Qbsr-11.1 loci with missense variants. *Ha16_00022871* showed the maximum mutation percentage in the *QBSR-16.1* locus followed by the *Ha16_00022789* and *Ha16_00022248* genes, all of which also harbored missense variants. The predominance of missense variants within these four QTL regions suggests that they are subject to positive selection pressure, indicating an evolutionary advantage in the context of SSR resistance.

### 3.4. Analysis of RGA Haplotype

To reveal the haplotype diversity of RGAs in different sunflower cultivars, we tested the gene-coding sequence (CDS) haplotype (gcHap) of our RGA candidates based on the SNPs described by Sariel Hübner et al. on the cultivated sunflower SNP database (2019) [50]. A total of 1013 out of the 1344 RGAs harbored at least three distinct major gcHaps, which have an average major gcHapN of 219.5 ± 108.7 (Table S4). No SNPs was found within the CDS regions of the remaining 331 RGAs, and were thus categorized as house-keeping (HK) genes, which are implicated in fundamental biological functions.

**Figure 4:**
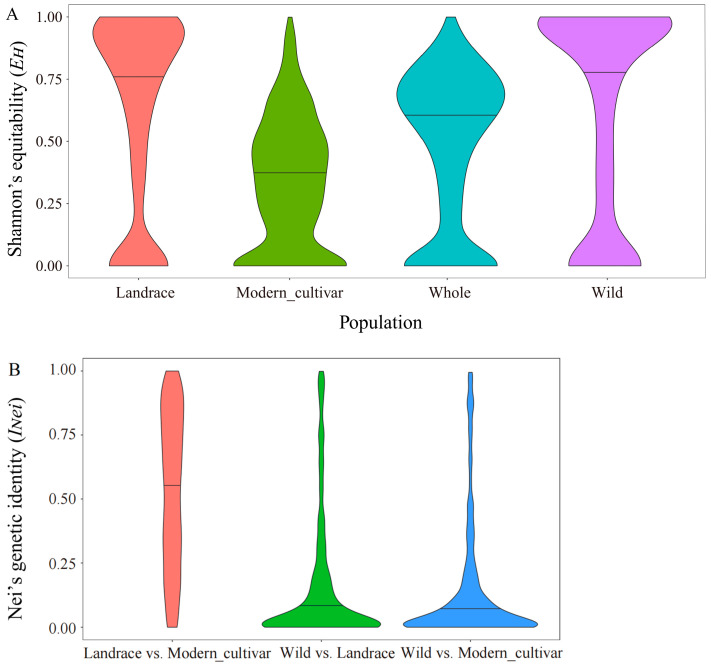

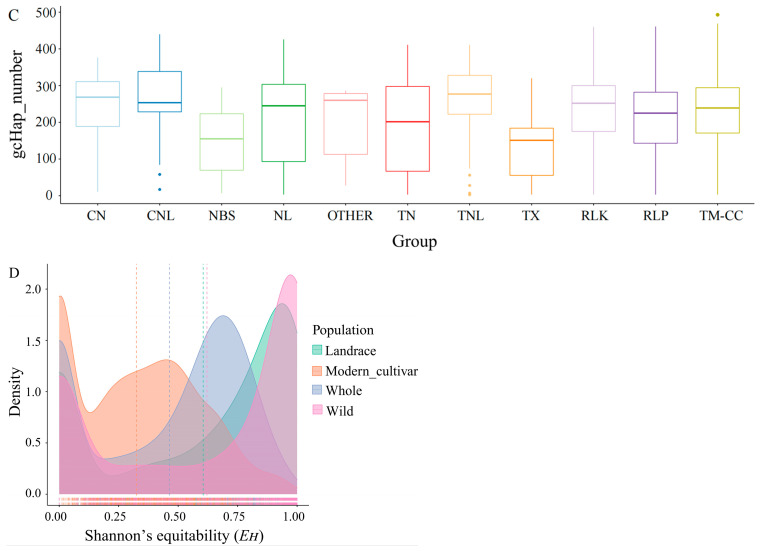
gcHap numbers, Shannon’s equitability (EH), and Nei’s genetic identity (INei) of sunflower RGA family among different populations: (**A**) EH distribution in four different populations; (**B**) INei distribution of landrace vs. modern_cultivar wild vs. modern_cultivar and wild vs. landrace; (**C**) distribution of gcHap number (gcHapN) of all 1013 RGA genes in different subgroups; (**D**) frequency distribution of EH in landrace, wild, modern_cultivar, and whole populations.

The gcHap number (gcHapN) varied considerably across sunflower chromosomes. Chromosome 9 exhibited the highest gcHapN with 19,147, followed by chromosomes 11 and 10 with 16,135 and 17,050, respectively. In contrast, chromosome 6 displayed the lowest gcHapN at 6377. By examining the gcHap diversity of 11 gene classes of 1013 RGAs, we found that gcHap number (gcHapN) was highest in CNL genes, followed by TNL genes, while TX genes had the lowest gcHapN. This suggests that CNL and TNL genes may have distinct roles across the 493 cultivars studied, and it also implies a greater stability in RLKs compared to CNLs and TNLs (Figure 4C). To understand the gcHap diversity of three major sunflower populations, we calculated the Shannon’s equability (EH) values within each varietal group using the gcHap data from all 1013 genes (Figure 4A,D). We found that genes in the wild cultivars had significantly higher mean gcHapN and EH than genes in other populations, whereas EH (gcHapN) was low for genes involved in modern cultivars (Figure 4B).

Additionally, we assessed the average Nei’s genetic identity (INei) among the populations. The average INei was smallest between wild and landrace, followed by that between wild and modern cultivar, and it was largest between landrace and modern cultivar. Generally, the lower the INei value of a gene, the greater its contribution to sunflower population differentiation, and vice versa.

### 3.5. RGA Gene Expression Responses to S. sclerotiorum

**Figure 5:**
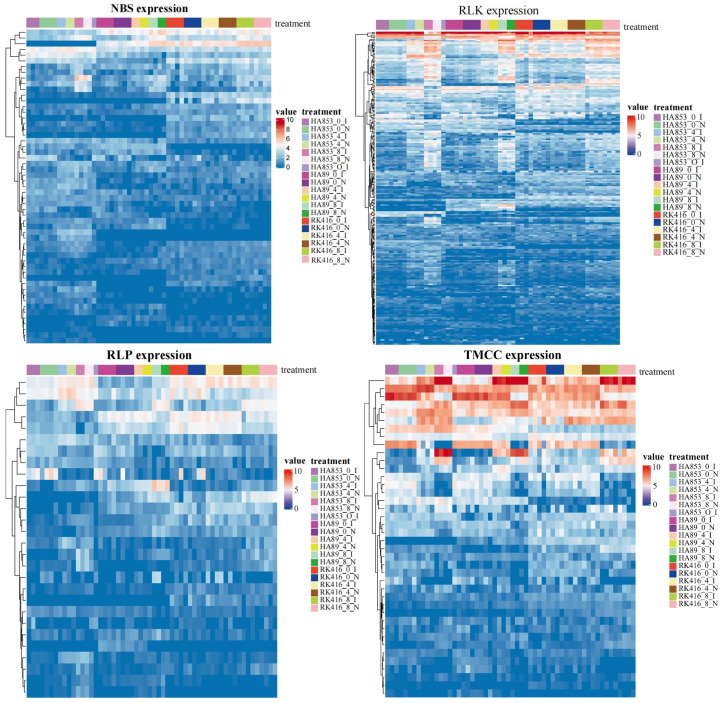
RGA gene expression responses to *S. sclerotiorum*: heat maps of differentially expressed RGA genes.

Sclerotinia head rot (SHR), caused by the necrotrophic fungus *S. sclerotiorum*, is one of the most destructive diseases of sunflower. Previous studies analyzed the expression patterns of one susceptible (HA89) and two tolerant sunflower ILs (HA853 and RK416) to SHR at 0, 4, and 8 days post inoculation (dpi) [3]. We expanded on this by conducting a comprehensive differential gene expression analysis across all time points for each IL, comparing the inoculated samples to the non-inoculated ones. Additionally, we examined the differential expression of genes (DEGs) between pairs of time points within each IL, as well as between pairs of ILs at identical time points under inoculated conditions, encompassing a total of twenty-seven unique combinations. A total of 348 differentially expressed RGA genes were found. The receptor-like kinase (RLK) family was predominant with 226 DEGs, followed by nucleotide-binding site (NBS) with 56 DEGs, and transmembrane coiled-coil (TM-CC) with 41 DEGs. Receptor-like proteins (RLPs) had only 29 DEGs. Notably, NBS and RLK showed low-expressed DEGs, while TM-CC showed the largest proportion of highly expressed DEGs (Figure 5).

The distribution of these 348 DEGs varied across the various combinations tested. Remarkably, only a single DEG was shared between the comparisons of HA853 at 0 days post inoculation (inoculated vs. non-inoculated) and HA853 at 8 days post inoculation (inoculated vs. non-inoculated), with no DEGs common to other combination pairs.

**Figure 6:**
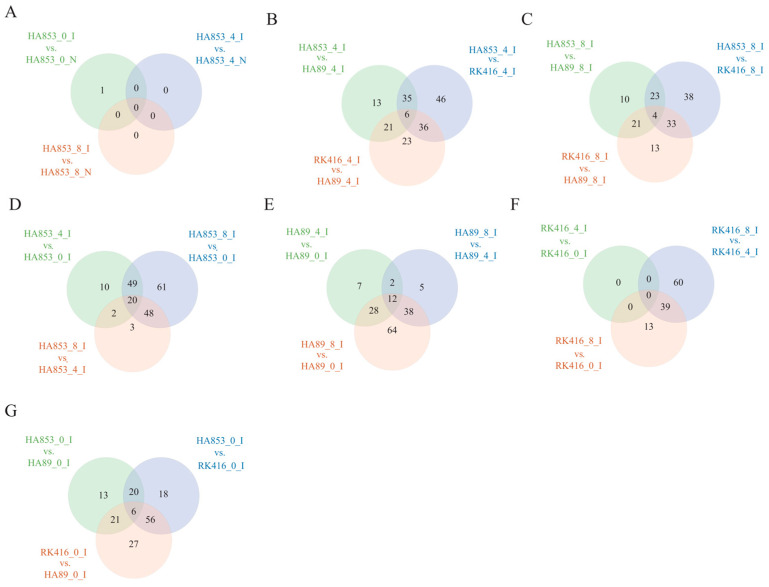
(**A**–**G**) differentially expressed RGA genes in the three datasets through Venn diagram software (available online: http://bioinformatics.psb.ugent.be/webtools/Venn/, accessed on 1 May 2022). Different colors mean different combinations.

In addition, the analysis of the DEG between time points within ILs showed a larger number of DEGs (8 dpi vs. 0 dpi) than those of 4 dpi vs. 0 dpi and 4 dpi vs. 8 dpi. These results indicated that the response of RGAs to SHR in the three cultivars was mainly in the late stage of the infection process. Additionally, the comparative analysis of DEGs among the IL time point combinations indicated variability in the number of DEG RGAs, with the comparison between HA853 and RK416 yielding the highest number of DEGs (Figure 6).

### 3.6. Functional Annotation and Enrichment of RGAs

**Figure 7:**
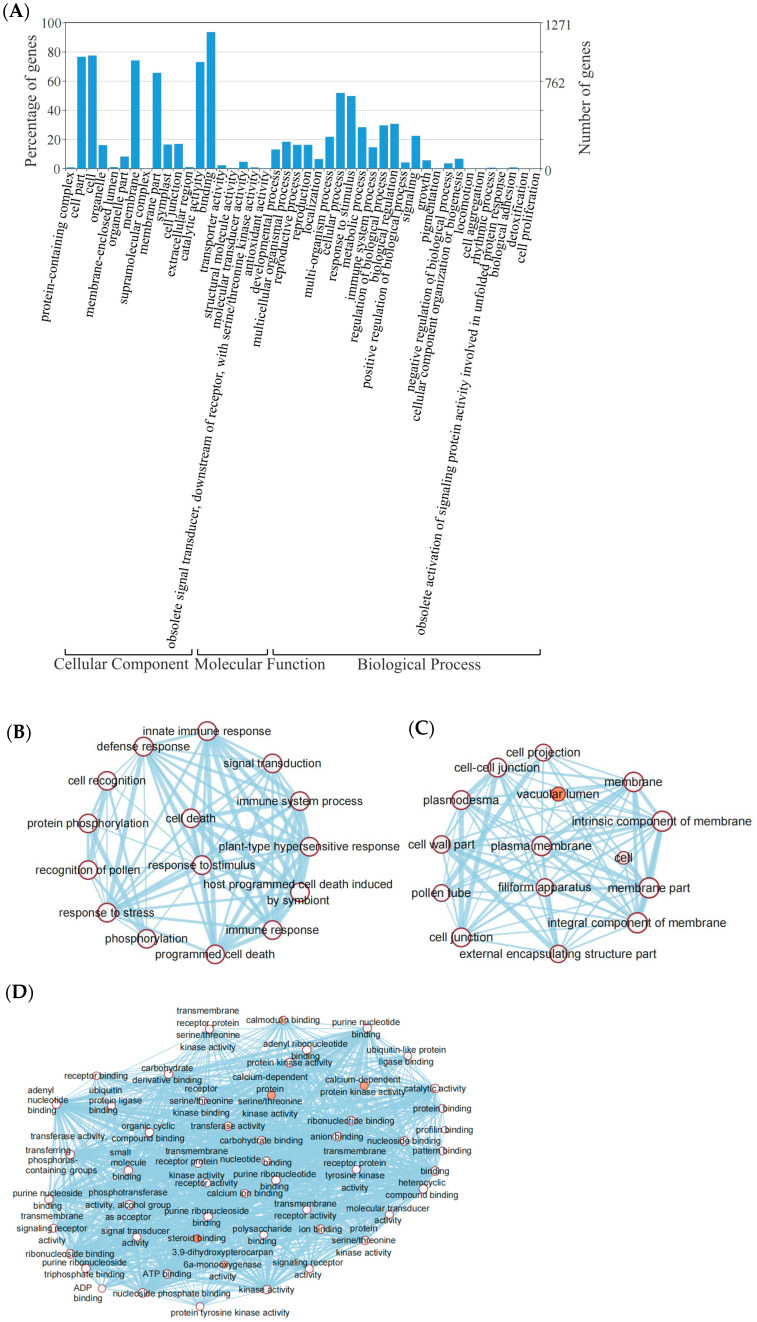
Gene ontology annotation of RGA and network diagram: (**A**) gene ontology annotation of RGAs. In total, 1344 sequences were grouped into 3 major functional categories and 44 sub-categories; (**B**) network diagram of GO terms enriched in biological process. Only the top 10% of GO terms with the lowest FDR (false discovery rate) value are shown; (**C**) network diagram of GO terms enriched in cellular component; (**D**) network diagram of GO terms enriched in molecular function. Nodes with type comments are white and others are orange.

To further understand the function of RGAs in organisms, we performed a gene ontology (GO) enrichment analysis of these genes. The results showed that RGAs were most frequently related to the GO terms of binding (GO:0005488), cell part (GO:0044464), and cell (GO:0005623) (Figure 7A). The enrichment analysis further revealed a significant overrepresentation of RGAs in numerous biological processes, particularly those GO terms associated with plant defense mechanisms, including defense response, response to stress, and response to stimulus. This is consistent with the function of RGAs in plant immunity (Figure 6A).

Moreover, the molecular functional analysis within the GO framework highlighted a substantial enrichment of RGAs in binding-related activities. Notable among these were GO terms for polysaccharide binding, protein binding and calcium ion binding, and profilin binding. The enrichment of these binding functions suggests a potential role for RGAs in resistance processes that are modulated by transcription factors, as illustrated in the network diagrams of GO terms enriched in biological processes (Figure 7B), cellular components (Figure 7C), and molecular functions (Figure 7D). These findings collectively support the hypothesis that RGAs contribute to the intricate network of plant defense responses, potentially through interactions with various molecular partners and signaling pathways.

### 3.7. Introgression of RGA Resistance Against S. sclerotiorum in Sunflower Cultivars

Previous studies have found that introgression from wild species has been a significant contributor to the genetic makeup of the cultivated sunflower pangenome, with approximately 10% of its genes derived from such events. Notably, among these introgressed genes, there were more genes related to biological resistance against *S. sclerotiorum* during sunflower breeding [50]. The RGA gene family plays an important role in plant resistance. Based on the results of Sariel Hübner et al. (2019) [50], we investigated whether there is introgression in RGAs. We found that introgression events appeared in 15 RGA genes. Specifically, the gene *Ha17_00009020* had the most extensive introgression in the SAM population, being involved in 35 cultivars and 5 neighborhood species (Table S5). It has been reported that introgression was related to the development of disease resistance against *S. sclerotiorum*, which is also related to downy mildew resistance [50]. In our case, the 15 RGAs identified with introgression events also offer considerable promise for future sunflower breeding, showing the potential to enhance disease resistance in cultivated varieties.

## 4. Discussion

Resistance (R) genes play a key role in plants’ remarkable innate immune system to recognize, or respond to, many pathogenic organisms. Multiple cloned R genes could be engineered into crops as a stack to avoid linkage drag and to delay emergence of virulent pathogens. In recent years, 16 rust resistance genes (*R1*–*R5*, *R10*–*R12*, *R13a*, *R13b*, *R14*, *Pu6*, *Radv*, *R15*, *R17*, and *R18*) and more than 40 downy mildew resistance genes (*Plv*–*Plz*, *Pl1*–*Pl35*, and *PlArg*) have been predicted in sunflower [30,31,32,33,34,35,36,37,38,39,40,41,42,43]. Clearly, comprehensive genomic profiling of the RGA genes offers invaluable insights into the evolutionary trajectory of R genes and their application in the enhancement of sunflower breeding programs [48,49]. However, the RGA candidate identification approach is restricted by the quality of the genome assembly. Pangenomes are references that capture the genetic repositories of a species rather than a single individual and can reduce reference bias in genomic analysis [54,55,56]. Therefore, the use of pangenome to identify candidate genes lays the pivotal groundwork for breeding of improved cultivars. To comprehensively identify the resistance gene resources of sunflower, we identified RGAs from the pangenome of sunflower and analyzed the distribution, domain structure, presence/absence, haplotype diversity, introgression, and SNPs of RGAs. We found that the largest class of RGA candidates was RLKs, which is consistent with observations in other plants such as cabbage, wild strawberry, and cotton [62,63]. This predominance of RLKs over NBS-LRR and RLP genes likely reflects their versatile functionality within the plant systems. The RLK gene has been reported to be involved in a variety of regulatory processes, such as interactions with symbionts, self-incompatibility, and the number of growth processes regulated in response to hormones [64]. However, other RGA classes such as NBS-LRR genes mainly focus on a resistance response that has lower functional diversity [65,66]. Leister (2004) reported that the over-representation of one of these families could reflect the adaptation of the R genes to the predominant pathogens [67].

We found considerable variation in RGA candidates’ content across the sunflower pangenome, with more than 82.4% of the pangenome classified as “dispensable” and “rare” genes. These results contrast with the *Brassica napus* and *Cucumis melo* pangenomes, in which 43.05% and 15% of the genes, respectively, were identified as dispensable [68,69].

We also found that the RGAs were unevenly distributed across the genomes and have also been reported in *Brassica oleracea* [70], rice [71], and other plants [69,72,73,74,75,76,77]. This uneven distribution may be due to recent tandem gene amplifications, segmental duplications, and dosage compensation. These genomic alterations may play a role in the adaptive responses of plants to their ever-changing environments and pathogenic challenges.

Several QTLs responsible for quantitative resistance have been identified in sunflower [45,48,78,79]. Sclerotinia resistance is a complex trait greatly affected by different environmental factors, such as temperature, humidity, and rainfall, so it is controlled by quantitative inheritance genes. Depending on the genome-wide association and bi-parental QTL mapping, more than one hundred QTLs were identified, contributing to *S. sclerotiorum* [45,48,80,81]. However, most of those detected relied on a single reference genome. In this context, we used a larger number of candidate genes derived from the sunflower pangenome. We identified 61 RGA candidates within 4 QTL regions associated with SSR resistance and presented on 4 chromosomes. The identification strategy of RGA candidates within QTLs could provide a basis for mapping candidate genes, which offers significant promise for the enhancement of sunflower breeding and germplasm improvement. Furthermore, the QTL-associated SNP markers described here could be transferred to breeding programs to accelerate the process of obtaining genotypes with better performance against SSR.

The gene-coding sequence haplotype (gcHap) was shown to be more available than SNPs for the detection of causal genes underlying complex diseases [60]. In this study, we characterized the gcHap diversity of 1344 RGA genes in 493 sunflower accessions. We observed that genes vary greatly in their gcHap diversity levels in different lines. Notably, wild cultivars exhibited a richer gcHap diversity compared to landrace and modern cultivars, indicating a reduction in RGA gene diversity due to selective breeding practices.

We also found the TX genes display low gcHap diversity, in which most of them may have undergone negative purifying selection during evolution. However, the CNL and TNL genes were more abundant across populations, potentially fulfilling diverse roles in plant disease resistance. We therefore conclude that gcHaps with appreciable frequencies in populations are the primary genetic basis for disease resistance diversity in plants. The comprehensive dataset of sunflower RGA gcHap diversity produced in this study could significantly advance research on sunflower resistance genes and contribute to the ongoing enhancement of disease resistance in future sunflower breeding efforts.

In total, 27 combinations of time points within three ILs upon infection revealed 348 DEGs. These DEGs were identified as potential SHR response genes. The expression pattern analysis of DEGs indicated that the TM-CC genes were up-regulated more often than other RGA classes, which suggested that TM-CC genes were more sensitive to *S. sclerotiorum*. In particular, sunflowers showed no visible SHR symptoms for at least ten days after inoculation, but microscopic lesions have been detected after a 24 h incubation period in susceptible and tolerant varieties. These early transcriptional shifts are indicative of the plant’s initial defense mechanisms. In this study, more DEGs were detected at 8 dpi vs. 0 dpi across all ILs, indicating that the RGAs play a significant role at early stages of defense, which has also been reported in other plant diseases. Indeed, the DEGs were unevenly distributed in all ILs, and more DEGs were detected between HA853 vs. RK416 than for other combinations, revealing different defense mechanisms between these two ILs. Moreover, 17 DEGs (Ha4_00025035, Ha11_00030769, Ha9_00012306, Ha16_00021249, Ha16_00021452, Ha16_00021682, Ha16_00021760, Ha16_00021825, Ha16_00022056, Ha16_00022073, Ha16_00022154, Ha16_00022246, Ha16_00022401, Ha16_00022602, Ha16_00022752, Ha16_00022793, and Ha16_00022884) were also detected within QTLs associated with SSR resistance. Among the 17 DEGs, 3 were selected to validate RNA-seq analysis using qPCR (Figure S1).

Gene ontology (GO) enrichment analysis showed that most RGAs were associated with defense processes.

Hübner et al. estimated that 10.6% of introgressions make up the SAM population. Our results provide further support for the contribution of RGA introgressions from wild species to disease resistance in cultivated sunflower. We also found that such introgressions increased the total number of RGAs in the sunflower pangenome.

The pangenome research of sunflower provides more abundant resources for sunflower resistance research, which fits previous findings in other plants such as *Melon* [68], *Brassica oleracea* [70], and *Brassica napus* [69]. The integration of genetic material from wild relatives into sunflower breeding is a sensible strategy aimed at enhancing resistance to environmental challenges and prevalent diseases, such as downy mildew, white mold, and rust. This approach offers the possibility of wild genetic diversity in the fortification of cultivated sunflower against biotic stresses.

## 5. Conclusions

In this study, we identified 1344 RGAs, of which 237 were core and 1107 were variable. Our results highlight the potential of variable genes to be used in genetic structural variation studies for future breeding programs. Moreover, our work revealed some novel RGAs that contribute to resistance to *S. sclerotiorum*. SNP-based haplotype analysis showed a greater diversity of RGA candidates in wild sunflower accessions compared to landraces and modern cultivars, underscoring the potential of wild genetic resources in breeding. It is expected that the core and dispensable genes, as well as the identified candidates with *S. sclerotiorum* resistance, will contribute to marker-assisted breeding in sunflower crops worldwide.

## Figures and Tables

**Table 1:** Counts of resistance gene classes screened from sunflower pangenome data.

RGAs	Reference	Pangenome Additional Contigs	Pangenome
CN	9 (3, 6)	0 (0, 0)	9 (3, 6)
CNL	27 (2, 25)	3 (2, 1)	30 (4, 26)
NBS	45 (14, 31)	0 (0, 0)	45 (14, 31)
NL	64 (14, 50)	0 (0, 0)	64 (14, 50)
RLK	752 (119, 633)	5 (1, 4)	757 (120, 637)
RLP	131 (29, 102)	0 (0, 0)	131 (29, 102)
TMCC	187 (19, 168)	4 (3, 1)	191 (22, 169)
TN	31 (4, 27)	1 (1, 0)	32 (5, 27)
TNL	41 (12, 29)	0 (0, 0)	41 (12, 29)
TX	38 (14, 24)	0 (0, 0)	38 (14, 24)
OTHER	6 (0, 6)	0 (0, 0)	6 (0, 6)
Total	1331 (230, 1101)	13 (7, 6)	1344 (237, 1107)

**Table 2:** Total number of RGAs across the 290 accessions on the reference genome, and pangenome additional contigs.

RGAs	Reference	Pangenome Additional Contigs	Pangenome
CN	304	0	304
CNL	274	10	284
NBS	2097	0	2097
NL	1975	0	1975
TN	941	258	1199
TNL	2475	0	2475
TX	2303	0	2303
RLK	14,974	48	15,022
RLP	4806	0	4806
TM-CC	2296	126	2422
Total	32,445	442	32,887

**Table 3:** The 61 RGAs within Qbsr-4.1, Qbsr-9.1, Qbsr-11.1, and Qbsr-16.1.

Locus	Pseudomolecule	Start (bp)	End (bp)	Length (bp)	RGA Candidate Classes	The Number of RGAs	Core Gene Percentage
Qbsr-4.1	Chr4	107,675,011	124,808,091	17,133,080	RLK 3, NBS 1, TM-CC 1	5	5 core (100% core)
Qbsr-9.1	Chr9	135,225,176	153,762,638	18,537,462	RLK 4, TM-CC 1	5	5 core (100% core)
Qbsr-11.1	Chr11	170,688,742	185,809,916	15,121,174	RLK 1	1	1 core (100% core)
Qbsr-16.1	Chr16	45,544,215	199,280,159	153,735,944	RLK 25, NBS 3, TM-CC 8, RLP 5, NL 4, CNL 2, TN 1,TX 1, OTHER 1	50	39 core (78% core)

## Data Availability

The data supporting the findings of this study are available within the article, its Supplementary Materials, or from the corresponding authors.

## Supplementary Materials

The following supporting information can be downloaded at https://www.mdpi.com/article/10.3390/life14101322/s1: Figure S1: Expression profiles of three selected RGA genes in response to *S. sclerotiorum*; Table S1: Primers used for RT-qPCR; Table S2: List of RGA genes of sunflower retrieved from the sunflower pan-genome database; Table S3: 290 accessions of sunflower used in this study; Table S4: The gcHap of RGAs; Table S5: The SAM population involved introgression from *H. argophyllus* and *H. petiolaris*, with wild *H. annuus*.
