# Supplementary material for: Pangenome Data Analysis Reveals Characteristics of Resistance Gene Analogs Associated with Sclerotinia sclerotiorum Resistance in Sunflower
Source: Life (Basel). 2024 Oct 17;14(10):1322. doi: 10.3390/life14101322 (PMC11509514; doi:10.3390/life14101322)
Supplement: Supplementary file 1 [file life-14-01322-s001.zip › Supplemental Figure S1.pdf]

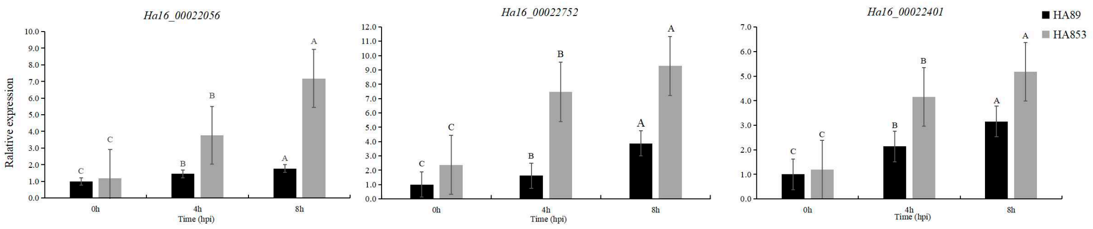

Supplemental Figure S1. Expression profiles of three selected RGA genes in response to *S. sclerotiorum*. The data are shown as the mean±standard error. Transcript levels were statistically analyzed by an ANOVA with Duncan's multiple range test (P<0.05) at one timepoint. Means in the plot followed by the same letter do not differ based on Duncan's multiple range test at P<0.05 (n=3).
