# Supplementary material for: Pangenome Data Analysis Reveals Characteristics of Resistance Gene Analogs Associated with Sclerotinia sclerotiorum Resistance in Sunflower
Source: Life (Basel). 2024 Oct 17;14(10):1322. doi: 10.3390/life14101322 (PMC11509514; doi:10.3390/life14101322)
Supplement: Supplementary file 1 [file life-14-01322-s001.zip › Supplemental Table S1.pdf]

Supplemental Table S1. Primers used for RT-qPCR

HanXRQ-Gene ID	HaRGA number	Primer sequence (5'-3')
HanXRQChr16g0505051	Ha16_00022752	GCCGGTTGTCAGCAGCATA
		AACAGCTTTCTTCGCACCAAA
HanXRQChr16g0515831	Ha16_00022056	AGCAGCTTGCGTGGTCAGA
		GGTGCAAACTGTTCCATTGTTG
HanXRQChr16g0506041	Ha16_00022401	TGTATAGATTGGGAGGGTGCAA
		CCTGGATTCTTCTTGGGATCAC
HanXRQChr14g0446641	Actin	CAATGTTCCCGCCATGTATG
		TGACACACCATCTCCAGAATCC
HanXRQChr13g0397831	Tubulin	GCACCGGTTCTGGTTTAGGA
		ACCTTTGGAGATGGGAATATGGT
